# Supplementary figures and images for: Subclinical articulatory changes of vowel parameters in Korean amyotrophic lateral sclerosis patients with perceptually normal voices
Source: PLoS One. 2023 Oct 13;18(10):e0292460. doi: 10.1371/journal.pone.0292460 (PMC10575489; doi:10.1371/journal.pone.0292460)

**S1 Fig. Association between ALSFRS-R speech subscore and vowel articulation index**

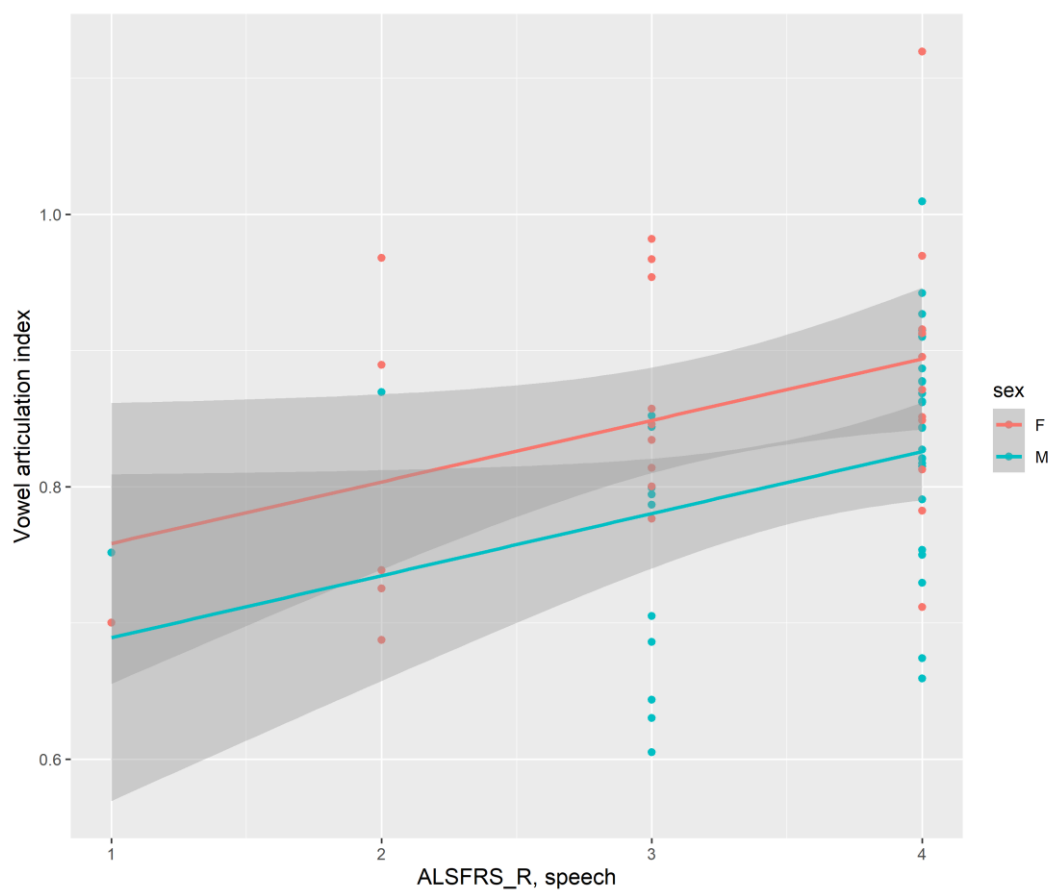

Supplement: S1 Fig — (PDF) [file pone.0292460.s001.pdf]
