# Supplementary material for: Subclinical articulatory changes of vowel parameters in Korean amyotrophic lateral sclerosis patients with perceptually normal voices
Source: PLoS One. 2023 Oct 13;18(10):e0292460. doi: 10.1371/journal.pone.0292460 (PMC10575489; doi:10.1371/journal.pone.0292460)

S2 Fig. Receiver operating characteristic curves for differentiating ALS patients with dysarthria from healthy controls using each vowel parameter

(A)

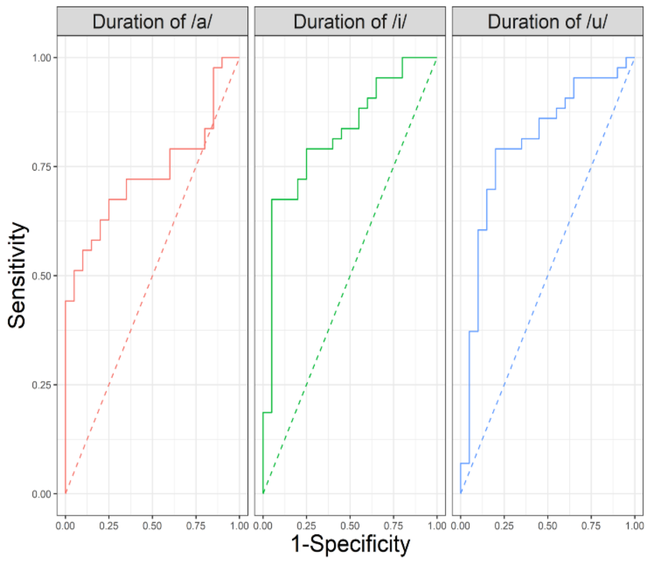

(B)

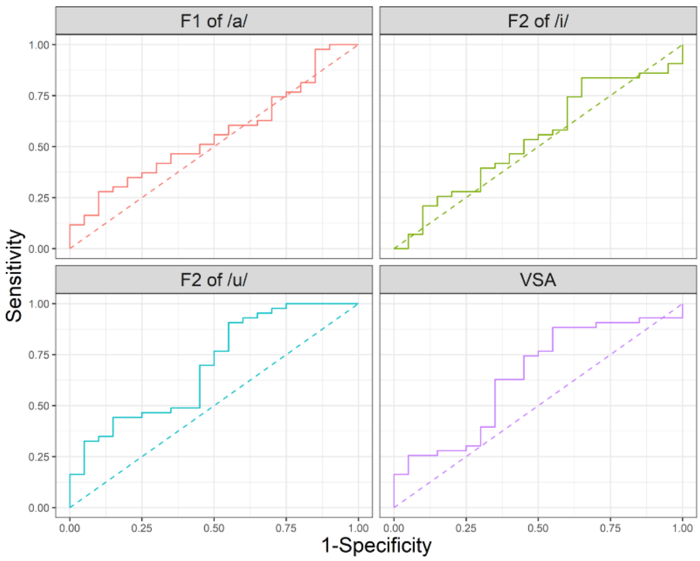

Supplement: S2 Fig — (PDF) [file pone.0292460.s002.pdf]
