# Supplementary material for: Subclinical articulatory changes of vowel parameters in Korean amyotrophic lateral sclerosis patients with perceptually normal voices
Source: PLoS One. 2023 Oct 13;18(10):e0292460. doi: 10.1371/journal.pone.0292460 (PMC10575489; doi:10.1371/journal.pone.0292460)
Supplement: S1 Table — (DOCX) [file pone.0292460.s003.docx]

**S1 Table. List of stimuli words**

| **Vowel** | i | u | a | | | |
| --- | --- | --- | --- | --- | --- | --- |
| **CV (6 repetitions)** | pi | pu | pa | | | |
| **Onset\Coda** | m | | | t | k | l |
| p | pim 빔 | pum 붐 | pam 밤 | pat 밭 | pak 박 | pal, p’al, p^h^al 발, 빨, 팔 |
| t | tim 딤 | tum 둠 | tam 담 | tat 닫 | tak 닥 | tal, t’al, t^h^al 달, 딸, 탈 |
| k | kim 김 | kum 굼 | kam 감 | kat 갓 | kak 각 | kal, k’al, k^h^al 갈, 깔, 칼 |
| s | sim 심 | sum 숨 | sam 삼 | sat 삿 | sak 삭 | sal, s’al 살, 쌀 |
| tɕ | tɕim 짐 | tɕum 줌 | tɕam 잠 | tɕat 잣 | tɕak 닥 | tɕal, tɕ’al, tɕ^h^al 잘, 짤, 찰 |

The three different types of consonants /t^h^, t, s/ (‘ㅌ, ㄷ, ㅅ’ in their Korean written forms) are pronounced as [t] in the coda position.
