# Supplementary material for: Subclinical articulatory changes of vowel parameters in Korean amyotrophic lateral sclerosis patients with perceptually normal voices
Source: PLoS One. 2023 Oct 13;18(10):e0292460. doi: 10.1371/journal.pone.0292460 (PMC10575489; doi:10.1371/journal.pone.0292460)
Supplement: S2 Table — (DOCX) [file pone.0292460.s004.docx]

**S2 Table. Effects of bulbar UMNs or LMNs on vowel parameters**

| **Parameter** | **None** | **UMNs only** | **LMNs only** | **UMNs+LMNs** | ***P* value** | |
| --- | --- | --- | --- | --- | --- | --- |
|  |  |  |  |  | **Overall** | **UMNs+LMNs vs. None** |
| Duration (ms) |  |  |  |  |  |  |
| /a/ | 79.6 (11.7) | 114.9 (42.1) | 122.6 (24.0) | 128.6 (32.8) | **.001** | **<.001** |
| /i/ | 101.2 (21.4) | 143.0 (76.6) | 185.5 (79.1) | 168.1 (38.1) | **.005** | **.003** |
| /u/ | 93.2 (23.0) | 131.6 (60.5) | 158.2 (45.5) | 156.7 (44.3) | **.004** | **.002** |
| F0 (Hz) |  |  |  |  |  |  |
| /a/ | 157.5 (25.2) | 165.2 (48.5) | 157.6 (33.8) | 153.1 (35.4) | .754 | NS |
| /i/ | 164.6 (28.7) | 167.5 (46.7) | 168.1 (63.2) | 152.9 (32.4) | .429 | NS |
| /u/ | 162.8 (27.7) | 169.3 (52.8) | 167.1 (56.1) | 156.4 (34.8) | .644 | NS |
| F1 (Hz) |  |  |  |  |  |  |
| /a/ | 757.3 (76.6) | 718.8 (87.0) | 776.4 (41.6) | 764.0 (99.0) | .263 | NS |
| /i/ | 402.6 (75.7) | 399.3 (62.8) | 384.3 (36.7) | 372.5 (58.3) | .507 | NS |
| /u/ | 407.9 (55.6) | 442.3 (66.4) | 533.2 (17.0) | 467.5 (86.9) | .104 | NS |
| F2 (Hz) |  |  |  |  |  |  |
| /a/ | 1389.3 (123.0) | 1327.0 (206.0) | 1463.8 (200.1) | 1427.4 (135.2) | .115 | NS |
| /i/ | 2053.2 (218.8) | 2060.5 (159.5) | 2201.0 (659.6) | 2012.7 (243.3) | .577 | NS |
| /u/ | 1112.4 (182.5) | 1169.6 (158.8) | 1632.7 (184.6) | 1343.8 (246.7) | **.006** | **.036** |
| Harmonics-to-noise ratio (dB) |  |  |  |  |  |  |
| /a/ | 7.9 (5.8) | 10.5 (2.7) | 7.5 (1.9) | 6.6 (5.7) | .360 | NS |
| /i | 14.5 (2.7) | 12.7 (6.4) | 14.2 (3.1) | 20.4 (3.0) | .190 | NS |
| /u/ | 12.7 (8.2) | 15.7 (5.6) | 14.2 (2.1) | 24.2 (4.5) | .432 | NS |
| Vowel space area (Hz^2^) | 166172.2 (71759.9) | 125818.9 (66979.6) | 97165.8 (99059.3) | 125370.4 (76742.0) | .220 | NS |
| Vowel articulation index (conventional unit) | 0.85 (0.07) | 0.83 (0.07) | 0.74 (0.16) | 0.77 (0.11) | .094 | NS |

Data are expressed as mean (standard deviation).

Significant findings with *P* < .050 are in **bold** fonts.

Abbreviations: UMNs, upper motor neuron sign; LMNs, lower motor neuron sign; NS, not significant.
