# Supplementary material for: Subclinical articulatory changes of vowel parameters in Korean amyotrophic lateral sclerosis patients with perceptually normal voices
Source: PLoS One. 2023 Oct 13;18(10):e0292460. doi: 10.1371/journal.pone.0292460 (PMC10575489; doi:10.1371/journal.pone.0292460)
Supplement: S3 Table — (DOCX) [file pone.0292460.s005.docx]

**S3 Table. Diagnostic performance of individual vowel parameters in distinguishing ALS patients with dysarthria from healthy controls**

|  | **Sensitivity** | **Specificity** | **PPV** | **NPV** | **AUC (95% CI)** |
| --- | --- | --- | --- | --- | --- |
| **Duration of /a/** | 0.512 | 0.950 | 0.957 | 0.475 | 0.733 (0.611-0.854) |
| **Duration of /i/** | 0.674 | 0.950 | 0.967 | 0.576 | 0.822 (0.713-0.931) |
| **Duration of /u/** | 0.791 | 0.800 | 0.895 | 0.640 | 0.800 (0.677-0.923) |
| **F1 of /a/** | 0.279 | 0.900 | 0.857 | 0.367 | 0.557 (0.407-0.707) |
| **F2 of /i/** | 0.837 | 0.350 | 0.735 | 0.500 | 0.538 (0.382-0.695) |
| **F2 of /u/** | 0.907 | 0.450 | 0.780 | 0.692 | 0.693 (0.547-0.839) |
| **VSA** | 0.884 | 0.450 | 0.776 | 0.643 | 0.643 (0.491-0.796) |

Abbreviations: ALS, amyotrophic lateral sclerosis; PPV, positive predictive value; NPV, negative predictive value; AUC, area under the curve; CI, confidence interval.
