# Supplementary material for: Subclinical articulatory changes of vowel parameters in Korean amyotrophic lateral sclerosis patients with perceptually normal voices
Source: PLoS One. 2023 Oct 13;18(10):e0292460. doi: 10.1371/journal.pone.0292460 (PMC10575489; doi:10.1371/journal.pone.0292460)
Supplement: S4 Table — (DOCX) [file pone.0292460.s006.docx]

**S4 Table. Comparisons of vowel parameters between groups in male subjects**

| **Parameter** | **ALSwD**  (n = 12) | **ALSwoD**  (n = 14) | **Control**  (n = 10) | ***P* value** | | | |
| --- | --- | --- | --- | --- | --- | --- | --- |
|  |  |  |  | **Overall** | **ALSwD**  **vs. ALSwoD** | **ALSwD**  **vs.**  **Control** | **ALSwoD vs.**  **Control** |
| Duration (ms) |  |  |  |  |  |  |  |
| /a/ | 120.5 (30.9) | 79.6 (14.1) | 77.1 (17.5) | **<.001** | **<.001** | **<.001** | 0.968 |
| /i/ | 169.2 (55.7) | 95.4 (21.2) | 111.9 (24.1) | **<.001** | **<.001** | **<.001** | 0.603 |
| /u/ | 158.2 (46.1) | 91.5 (21.1) | 99.0 (28.9) | **<.001** | **<.001** | **<.001** | 0.737 |
| F0 (Hz) |  |  |  |  |  |  |  |
| /a/ | 142.0 (26.1) | 132.1 (23.9) | 128.5 (15.4) | .300 | NS | NS | NS |
| /i/ | 139.9 (22.9) | 137.3 (24.1) | 128.6 (17.8) | .427 | NS | NS | NS |
| /u/ | 140.4 (23.2) | 135.8 (23.5) | 130.3 (16.2) | .516 | NS | NS | NS |
| F1 (Hz) |  |  |  |  |  |  |  |
| /a/ | 700.3 (64.8) | 677.9 (32.5) | 748.2 (37.3) | **.008** | .288 | .068 | **.007** |
| /i/ | 355.6 (44.5) | 373.5 (57.5) | 367.6 (56.5) | .713 | NS | NS | NS |
| /u/ | 462.9 (86.9) | 435.5 (68.6) | 418.1 (48.3) | .337 | NS | NS | NS |
| F2 (Hz) |  |  |  |  |  |  |  |
| /a/ | 1322.6 (120.5) | 1276.2 (95.0) | 1315.5 (91.6) | .544 | NS | NS | NS |
| /i/ | 1910.6 (108.3) | 1925.5 (154.6) | 2005.4 (191.4) | .500 | NS | NS | NS |
| /u/ | 1387.2 (287.5) | 1147.0 (185.3) | 1072.0 (196.6) | **.006** | **.041** | **.009** | .752 |
| Harmonics-to-noise ratio (dB) |  |  |  |  |  |  |  |
| /a/ | 7.7 (3.5) | 8.0 (5.5) | 9.9 (2.2) | .466 | NS | NS | NS |
| /i/ | 6.3 (17.9) | 14.3 (3.0) | 14.6 (2.1) | .119 | NS | NS | NS |
| /u/ | 7.3 (24.0) | 14.9 (3.3) | 16.0 (2.0) | .285 | NS | NS | NS |
| Vowel space area (Hz^2^) | 93723.6 (47440.1) | 99693.7 (52584.1) | 165302.7 (64573.6) | **.007** | .959 | **.009** | **.025** |
| Vowel articulation index (conventional unit) | 0.75 (0.09) | 0.81 (0.07) | 0.87 (0.09) | **.008** | .237 | **.006** | .241 |

Data are expressed as mean (standard deviation).

Significant findings with *P* < .050 are in **bold** fonts.

Abbreviations: ALS, amyotrophic lateral sclerosis; ALSwD, ALS with dysarthria; ALSwoD, ALS without dysarthria; NS, not significant.
