# Supplementary material for: Subclinical articulatory changes of vowel parameters in Korean amyotrophic lateral sclerosis patients with perceptually normal voices
Source: PLoS One. 2023 Oct 13;18(10):e0292460. doi: 10.1371/journal.pone.0292460 (PMC10575489; doi:10.1371/journal.pone.0292460)
Supplement: Attachment — Submitted filename: Response to Reviewers.docx [file pone.0292460.s009.docx]

**General Comments for the Reviewers and the Editors:**

First, we sincerely thank the anonymous reviewers, the Associate Editor, and the Editor-in-Chief for their valuable comments. We have carefully addressed the reviewers’ observations and suggestions and revised our paper accordingly. Detailed responses to the reviewers’ comments are provided below. The original reviewer’s comments are in black, and our responses are provided in blue. The appropriate changes in the annotated manuscript (PONE-D-23-09560) are highlighted in yellow.

**Review Comments to the Author:
Reviewer #1**

General Summary
Thank you for the opportunity to review a manuscript titled "Subclinical articulatory changes of vowel parameters in Korean amyotrophic lateral sclerosis patients with perceptually normal voices." In the study, the authors strive to provide a pilot description of vowel articulation deficits in amyotrophic lateral sclerosis (ALS); for the study, authors recorded consonant-vowel (CV) and consonant-vowel-consonant (CVC) utterances from 43 patients diagnosed with definite, probable, or possible ALS and 20 healthy control (HC) participants. The authors found a significant increase in the length of the corner vowels and alterations of the first two format frequencies, as well as the vowel space area (VSA). The authors also performed a classification experiment reaching the area under the curve (AUC) of 0.912.
Generally, authors provide scientifically sound manuscripts with the most detailed descriptions of their experiments. The study design shows few shortcomings, especially considering the dataset; however, authors strive to deal with the issues when possible and discuss possible impacts of the study's limitations.

Suggestions and questions
1) The authors performed a classification experiment, however, reaching interesting AUC and also performed the linear regression analysis; thus, I would expect that the classification experiment was based on logistic regression, but as it is not clearly stated, it may not be apparent and suggest including methodology part where authors describe the classification scenario a validation method.

**Response:** We appreciate your valuable comments. We conducted ROC curve analysis using logistic regression with a generalized linear model. To provide further clarity, we have included a more detailed explanation of the ROC curve analysis in the methods section of the revised manuscript.

**Revised:** The diagnostic performance of selected vowel parameters was assessed by receiver operating characteristic (ROC) curve analysis using logistic regression with a generalized linear model.

2) The major weakness of the study is the age and sex match of HC participants. The authors strived to remove the effect of age and sex in the statistical analysis and included them in the linear regression analysis. Moreover, the authors acknowledge the possible confounding effect of sex differences in the limitations. Even though the age difference did not reach the significance level, it is the border value with p = 0.08, and therefore it would be beneficial to discuss it also. Especially because the HC group is younger and thus be advantaged in the analysis. Authors should discuss the results of their linear regression in that manner.

**Response:** We agree with your opinion. In order to match the ages between the disease and control groups, we enrolled an equal number of participants in each decade. However, we acknowledge that patients with ALS seemed to be slightly older than the HCs, and thus may have had a potential advantage in the analysis. We have addressed this point in the discussion of the revised manuscript.

**Added:** While not statistically significant, we acknowledge that patients with ALS were slightly older than the HCs, potentially conferring an advantage in the analysis. Nevertheless, we anticipate that our findings will remain unaffected by potential age differences for the following reasons: Regarding the formant frequencies of the corner vowels, a recent study revealed a decline in F2 /a/ and F2 /u/ with age, while F1 remained unchanged^1^. However, our study showed that F1 /a/ and F2 /i/ decreased, while F2 /u/ rather increased in older ALS patients compared to HCs, showing contradictory results on F2 /u/. Furthermore, some studies reported that the formant frequencies of the corner vowels change minimally across several decades of adult life, with no significant differences observed between middle-aged women (40 to 60 years) and older women (70 to 92 years)^2,3^. Therefore, the 5.5-year difference between ALSwD and HCs, as well as the 8-year difference between ALSwoD and HCs, is unlikely to impact our results on formant frequencies. Additionally, the VSA is known to show a compensatory increase with age^1^. However, in our study, we observed that VSA rather decreased in relatively older ALS patients compared to HCs, supporting our results of a reduced VSA inherent to the disease. Lastly, vowel durations exhibit a slight increase of no more than 30 ms with age, particularly in individuals aged over 70 years^1^. However, when comparing the significant differences in vowel durations between patients with ALS and HCs in our study—exceeding 50 ms for all corner vowels—it becomes evident that vowel durations are longer in patients with ALS, even after adjusting for age differences.

**References:**

1. Tykalova T, Skrabal D, Boril T, Cmejla R, Volin J, Rusz J. Effect of Ageing on Acoustic Characteristics of Voice Pitch and Formants in Czech Vowels. J Voice. 2021;35(6):931.e21-931.e33.

2. Eichhorn JT, Kent RD, Austin D, Vorperian HK. Effects of Aging on Vocal Fundamental Frequency and Vowel Formants in Men and Women. J Voice. 2018;32(5):644.e1-644.e9.

3. Albuquerque L, Oliveira C, Teixeira A, Sa-Couto P, Figueiredo D. [1]. J Voice. 2023;37(1):143.e13-143.e29.

3) How was the measurement of intensity standardized? If I understand correctly, participants were recorded directly by the device, not by the for-instance head-mounted microphone.

**Response:** We appreciate your critical comments. As you pointed out, we recorded participants' voices directly without using a head-mounted microphone, making it challenging to standardize vowel intensity in our recording setting. Therefore, we have decided to completely remove the intensity parameter from the manuscript. We have described this limitation in the discussion of the revised manuscript.

**Added:** Third, as we recorded participants' voices directly without using a head-mounted microphone, standardizing the vowel intensity in our recording setting became challenging. Consequently, we excluded the vowel intensity parameter from the analysis.

4) What are units of harmonic-to-noise ratio? If it is the Praat value, I would expect decibels, but the values listed in the results are low. It could be because the authors recorded lower sound quality by the device. If this is true, I would suggest the speech recording guideline, which also provides recording setup information. (Rusz, J., Tykalova, T., Ramig, L. O., & Tripoliti, E. (2021). Guidelines for speech recording and acoustic analyses in dysarthrias of movement disorders. Movement Disorders, 36(4), 803-814.)

**Response:** As a type of objects in *Praat*, harmonicity (with cross-correlation method), the unit of harmonics-to-noise ratio is dB. To clarify, we have added the units of each vowel parameter in Table 2 and within the definition of each parameter in the methods section (speech analysis chapter) of the revised manuscript. The analyzed speech samples were recorded in a quiet room using a voice recorder (ICD-PX370 Mono Digital Voice Recorder, Sony Corporation, Tokyo, Japan), but the recording quality appears to suffer because participants were recorded directly by the device without using a head-mounted microphone and the patients' voice intensity was usually low. For the follow-up study, we will adhere to the speech recording guidelines you suggested. Thank you.

**Added:** In addition, it is important to interpret our results on the harmonics-to-noise ratio with caution due to potential recording quality issues resulting from noise disturbances caused by the absence of a head-mounted microphone and low voice intensity.

5) What was the recording setup? What was the sampling frequency and level of quantization?

**Response:** The speech recording was not performed within a soundproof room, and we did not use a head-mounted microphone. Instead, we collected speech samples using a voice recorder in a quiet room. We have described this limitation in the revised manuscript. The sampling frequency was 44.1 kHz, and the quantization level was set to 16 bits.

**Added:** The sampling frequency was 44.1 kHz, and the quantization level was set to 16 bits.

6) Why authors did not include Vowel Articulation Index?

**Response:** We sincerely appreciate your valuable suggestion. The vowel space area (VSA) is a useful metric for studying speech production deficits and reductions in intelligibility, which is why we chose to utilize it in our study^1^. However, we are aware that the vowel articulation index (VAI) has been developed to mitigate interspeaker variability, although it is known to be sensitive to vowel formant centralization^2^. Taking your suggestion into consideration, we calculated each speaker's VAI as (F2i+F1a)/(F2u+F2a+F1u+F1i) and conducted the same statistical tests with the other vowel parameters. The findings of the VAI analysis were added to the results section, Table 2, and Table 3 in the revised manuscript.

**Added #1:** The VAI is designed to be as insensitive to interspeaker variability as possible while being as sensitive as possible to vowel formant centralization. Therefore, we additionally calculated each speaker’s VAI as (F2 of /i/ + F1 of /a/) / (F2 of /u/ + F2 of /a/ + F1 of /u/ + F1 of /i/).

**Added #2:** The VAI was significantly lower in ALSwD compared to HCs, and it also showed a slight reduction, though not statistically significant, in ALSwoD compared to HCs.

**Added #3:** The VAI decreased along with the ALSFRS-R speech subscore, as did the VSA.

**References:**

1. Sandoval S, Berisha V, Utianski RL, Liss JM, Spanias A. Automatic assessment of vowel space area. J Acoust Soc Am. 2013;134(5):EL477-83.

2. Caverlé MWJ, Vogel AP. Stability, reliability, and sensitivity of acoustic measures of vowel space: A comparison of vowel space area, formant centralization ratio, and vowel articulation index. J Acoust Soc Am. 2020;148(3):1436.

7) At the end of the page, 14 authors discuss the speech rate; they may find interesting studies
a. Novotny, M., Melechovsky, J., Rozenstoks, K., Tykalova, T., Kryze, P., Kanok, M., ... & Rusz, J. (2020). Comparison of automated acoustic methods for oral diadochokinesis assessment in amyotrophic lateral sclerosis. Journal of Speech, Language, and Hearing Research, 63(10), 3453-3460.
b. Rong, P. (2020). Automated acoustic analysis of oral diadochokinesis to assess bulbar motor involvement in amyotrophic lateral sclerosis. Journal of Speech, Language, and Hearing Research, 63(1), 59-73.

**Response:** We appreciate your thoughtful comment. As reported in previous studies, individuals with ALS exhibit a slower speaking rate (words per minute) and reduced speech segment duration (sec) compared to HCs^1^. These measurements can be influenced by cognitive impairment. However, in order to minimize the impact of cognitive dysfunction in patients with ALS, we employed structured speech stimuli consisting of single distinct syllables and 6 repetitions of syllables such as 'pa, pi, pu'. The latter can also be utilized for evaluating oral diadochokinesis (DDK)^2,3^. Nevertheless, it is important to clarify that the current study specifically focuses on articulatory changes of vowel parameters and does not involve the assessment of oral DDK. We plan to address this topic in a follow-up study. Thank you.

**References:**

1. Nevler N, Ash S, McMillan C, Elman L, McCluskey L, Irwin DJ, et al. Automated analysis of natural speech in amyotrophic lateral sclerosis spectrum disorders. Neurology. 2020;95(12):e1629-e1639.

2. Novotny, M., Melechovsky, J., Rozenstoks, K., Tykalova, T., Kryze, P., Kanok, M., et al. Comparison of automated acoustic methods for oral diadochokinesis assessment in amyotrophic lateral sclerosis. J Speech, Lang Hear Res. 2020;63(10):3453-3460.

3. Rong P. Automated Acoustic Analysis of Oral Diadochokinesis to Assess Bulbar Motor Involvement in Amyotrophic Lateral Sclerosis. J Speech Lang Hear Res. 2020;63(1):59-73.

Conclusion
To conclude, the author's scientifically sound, comprehensively written manuscript on an exciting topic. The authors acknowledge that this is a pilot study with several shortcomings. The study still needs improvement before it can be published, and some of the modifications can be timely. Therefore I suggest a major revision of the manuscript before publication.

**Reviewer #2**

The manuscript aims to characterize vowel properties in Korean ALS patients, investigate associations between vowel parameters and clinical features of ALS, and analyze subclinical articulatory changes of vowel parameters in those with perceptually normal voices. In general, the article is well written with good scientific novelty. However, some methodological issues need to be clarified, including the unbalanced age of healthy control participants. The motivation of study also needs to be improved.

**Abstract:**

Please add the information that patients were separated into dysarthria and without dysarthria groups based on ALS Functional Rating Scale-Revised speech item.

**Response:** Thank you for the comment. As per the reviewer’s suggestion, we have added information on the criteria for determining the presence of dysarthria based on the ALSFRS-R speech subscores in the revised manuscript.

**Revised:** Forty-three patients with ALS (27 with dysarthria and 16 without dysarthria) and 20 healthy controls were prospectively collected in the study. Dysarthria was assessed using the ALS Functional Rating Scale-Revised (ALSFRS-R) speech subscores, with any loss of 4 points indicating the presence of dysarthria.

**Introduction:**

The motivation of the study needs to be improved. I would suggest going beyond the ALS studies and improving the motivation based on other motor speech disorders. For example, the possibility of detecting the progression of the disease or subclinical articulatory changes has already been shown in Parkinson's disease patients with no perceptible dysarthria or even in patients with REM sleep behaviour disorder. See studies:

Skrabal D, Rusz J, Novotny M, Sonka K, Ruzicka E, Dusek P, Tykalova T. Articulatory undershoot of vowels in isolated REM sleep behavior disorder and early Parkinson's disease. NPJ Parkinsons Dis. 2022 Oct 20;8(1):137. doi: 10.1038/s41531-022-00407-7. PMID: 36266347; PMCID: PMC9584921.

Rusz J, Cmejla R, Tykalova T, Ruzickova H, Klempir J, Majerova V, Picmausova J, Roth J, Ruzicka E. Imprecise vowel articulation as a potential early marker of Parkinson's disease: effect of speaking task. J Acoust Soc Am. 2013 Sep;134(3):2171-81. doi: 10.1121/1.4816541. PMID: 23967947.

Skodda S, Grönheit W, Schlegel U. Impairment of vowel articulation as a possible marker of disease progression in Parkinson's disease. PLoS One. 2012;7(2):e32132. doi: 10.1371/journal.pone.0032132. Epub 2012 Feb 28. PMID: 22389682; PMCID: PMC3289640.

**Response:** We greatly appreciate your valuable suggestion. In response, we have expanded the motivation of our study beyond ALS-related research by referencing the recommended papers.

**Added:** Impairment of vowel articulation has been identified as a potential surrogate marker for neurodegenerative disorders, such as Parkinson's disease (PD), and can even be observed in individuals with rapid eye movement sleep behavior disorder, which is considered a prodromal stage of PD^1,2^. This impairment may worsen as the disease progresses^3^. However, there is a lack of studies specifically investigating vowel articulatory changes in patients with ALS.

**References:**

1. Skrabal D, Rusz J, Novotny M, Sonka K, Ruzicka E, Dusek P, et al. Articulatory undershoot of vowels in isolated REM sleep behavior disorder and early Parkinson's disease. NPJ Parkinsons Dis. 2022;8(1):137.

2. Rusz J, Cmejla R, Tykalova T, Ruzickova H, Klempir J, Majerova V, et al. Imprecise vowel articulation as a potential early marker of Parkinson's disease: effect of speaking task. J Acoust Soc Am. 2013;134(3):2171-81.

3. Skodda S, Grönheit W, Schlegel U. Impairment of vowel articulation as a possible marker of disease progression in Parkinson's disease. PLoS One. 2012;7(2):e32132.

**Methods:**

Speech collection: Please clarify how many occurrences (words) for each corner vowel /a/, /i/ or /u/ were used for statistical analyses. Were all these values used for final statistical analyses or only the average value (median value) for each parameter and corner vowel?

**Response:** In this pilot study, each participant was given 6 words for /i/, 6 words for /u/, and 30 words for /a/. For the subsequent study examining the effects of pre- and post-vocalic consonants on the production of non-high vowels, more /a/ words were recorded compared to /i/ and /u/ words. As shown in the revised Table S1, each participant produced 11 instances of /i/, 11 instances of /u/, and 35 instances of /a/, since CV words such as 'pa, pi, pu' were repeated 6 times each. We have included this missing information regarding CV repetitions in the revised Table S1. For the statistical analyses, the average values of each participant's vowels were used.

Speech analysis: Since the calibration of microphone for loudness was not performed, the measurement if intensity cannot provide valid results. Please delete this measurement from the whole article.

**Response:** We appreciate your thoughtful suggestion. Taking into consideration the absence of a head-mounted microphone and the lack of loudness calibration, we have completely removed the intensity parameter from the manuscript. We have also described this limitation in the discussion of the revised manuscript.

**Added:** Third, as we recorded participants' voices directly without using a head-mounted microphone, standardizing the vowel intensity in our recording setting became challenging. Consequently, we excluded the vowel intensity parameter from the analysis.

**Results:**

Please add units for each acoustic measurement used in Table 2 or within the definition of each measurement in the speech analyses chapter.

**Response:** Accordingly, we have added the units of each vowel parameter in Table 2 and within the definition of each parameter in the methods section (speech analysis chapter) of the revised manuscript.

**Discussion:**

Why was the younger group of healthy controls used?

The using of ANOVA test here is not representative. The t-test between healthy controls and ALS without dysarthria is likely to be significant based on presented median values: ALS with dysarthria (median age value 60.0 [25th percentile 50.0; 75th percentile 66.0] years), ALS without dysarthria 62.5 [48.5;67.0], healthy controls 54.5 [42.0;60.5]. The age-dependence of many acoustic measures, including formants and vowel space area has been demonstrated in previous studies even in healthy controls. See for example:

Kent RD, Vorperian HK. Static measurements of vowel formant frequencies and bandwidths: A review. J Commun Disord. 2018 Jul-Aug;74:74-97. doi: 10.1016/j.jcomdis.2018.05.004. Epub 2018 Jun 1. PMID: 29891085; PMCID: PMC6002811.

Tykalova T, Skrabal D, Boril T, Cmejla R, Volin J, Rusz J. Effect of Ageing on Acoustic Characteristics of Voice Pitch and Formants in Czech Vowels. J Voice. 2021 Nov;35(6):931.e21-931.e33. doi: 10.1016/j.jvoice.2020.02.022. Epub 2020 Mar 31. PMID: 32245663.

Therefore, I recommend authors to recruit a better age-matched healthy control group of participants or at least to improve discussion based on results in the study published by Tykalova T et al. (2021). This study is based on a large sample of healthy controls of both genders and covers the same acoustic measurements, including f0, F1, F2, VSA and vowel duration.

**Response:** We sincerely appreciate your constructive comments. While we enrolled an equal number of participants in each decade to match the ages between the disease and control groups, we acknowledge that patients with ALS seemed to be slightly older than HCs, and this age difference may have provided an advantage in the analysis. In response to your suggestion, we referenced the recommended papers and incorporated additional discussions on this issue in the revised manuscript.

**Added:** While not statistically significant, we acknowledge that patients with ALS were slightly older than the HCs, potentially conferring an advantage in the analysis. Nevertheless, we anticipate that our findings will remain unaffected by potential age differences for the following reasons: Regarding the formant frequencies of the corner vowels, a recent study revealed a decline in F2 /a/ and F2 /u/ with age, while F1 remained unchanged^1^. However, our study showed that F1 /a/ and F2 /i/ decreased, while F2 /u/ rather increased in older ALS patients compared to HCs, showing contradictory results on F2 /u/. Furthermore, some studies reported that the formant frequencies of the corner vowels change minimally across several decades of adult life, with no significant differences observed between middle-aged women (40 to 60 years) and older women (70 to 92 years)^2,3^. Therefore, the 5.5-year difference between ALSwD and HCs, as well as the 8-year difference between ALSwoD and HCs, is unlikely to impact our results on formant frequencies. Additionally, the VSA is known to show a compensatory increase with age^1^. However, in our study, we observed that VSA rather decreased in relatively older ALS patients compared to HCs, supporting our results of a reduced VSA inherent to the disease. Lastly, vowel durations exhibit a slight increase of no more than 30 ms with age, particularly in individuals aged over 70 years^1^. However, when comparing the significant differences in vowel durations between patients with ALS and HCs in our study—exceeding 50 ms for all corner vowels—it becomes evident that vowel durations are longer in patients with ALS, even after adjusting for age differences.

**References:**

1. Tykalova T, Skrabal D, Boril T, Cmejla R, Volin J, Rusz J. Effect of Ageing on Acoustic Characteristics of Voice Pitch and Formants in Czech Vowels. J Voice. 2021;35(6):931.e21-931.e33.

2. Eichhorn JT, Kent RD, Austin D, Vorperian HK. Effects of Aging on Vocal Fundamental Frequency and Vowel Formants in Men and Women. J Voice. 2018;32(5):644.e1-644.e9.

3. Albuquerque L, Oliveira C, Teixeira A, Sa-Couto P, Figueiredo D. A Comprehensive Analysis of Age and Gender Effects in European Portuguese Oral Vowels. J Voice. 2023;37(1):143.e13-143.e29.
